# A critical reflection on the principles of peroxisome formation in yeast

**DOI:** 10.3389/fphys.2014.00110

**Published:** 2014-03-20

**Authors:** Marten Veenhuis, Ida J. van der Klei

**Affiliations:** Molecular Cell Biology, University of GroningenGroningen, Netherlands

**Keywords:** peroxisome, fission, endoplasmic reticulum, *de novo* peroxisome formation, yeast

## Abstract

We have evaluated the current knowledge on peroxisome proliferation in yeast. In wild-type cells, peroxisomes multiply predominantly by fission at conditions that require peroxisome function(s) for growth. In cells that lack peroxisomes, for instance in *pex3* and *pex19* mutants or in mutants that display inheritance defects, peroxisomes may form *de novo*. We propose a novel machinery for the *de novo* formation of peroxisomes in *pex3* cells, in which new peroxisomes do not arise from the endoplasmic reticulum. This machinery is based on the recent observation that membrane vesicles are present in *pex3* cells that display peroxisomal characteristics in that they contain specific peroxisomal membrane and matrix proteins. These structures are the source for newly formed peroxisomes upon reintroduction of Pex3. Furthermore, we critically evaluate the principles of sorting of other peroxisomal membrane proteins to their target organelle and the function of the endoplasmic reticulum therein.

## Introduction

Peroxisomes are highly versatile organelles that readily adapt their numbers and physiological function in relation to metabolic needs. This functional flexibility requires a careful regulation of controlling organelle number and size. The factors controlling organelle size are still an enigma.

In yeast, low numbers of peroxisomes are normally present in cells grown at glucose excess conditions. However, when the cells are placed in media supplemented with carbon sources that require peroxisomal enzymes for growth (i.e., fatty acids, methanol, purines, and D-amino acids), organelle proliferation rapidly starts (Veenhuis et al., 1978). The mode of yeast peroxisome multiplication is still controversial. The current models range from the suggestion that in normal wild-type (WT) cells peroxisome multiplication exclusively results from fission to the view that all organelles form *de novo* from the endoplasmic reticulum (ER). Also combinations of these two modes have been suggested.

This contribution presents a critical overview of recent data on peroxisome multiplication in yeast and proposes possible novel directions aimed at resolving the molecular mechanisms of peroxisome formation.

## Peroxisome development

**Figure 1:**
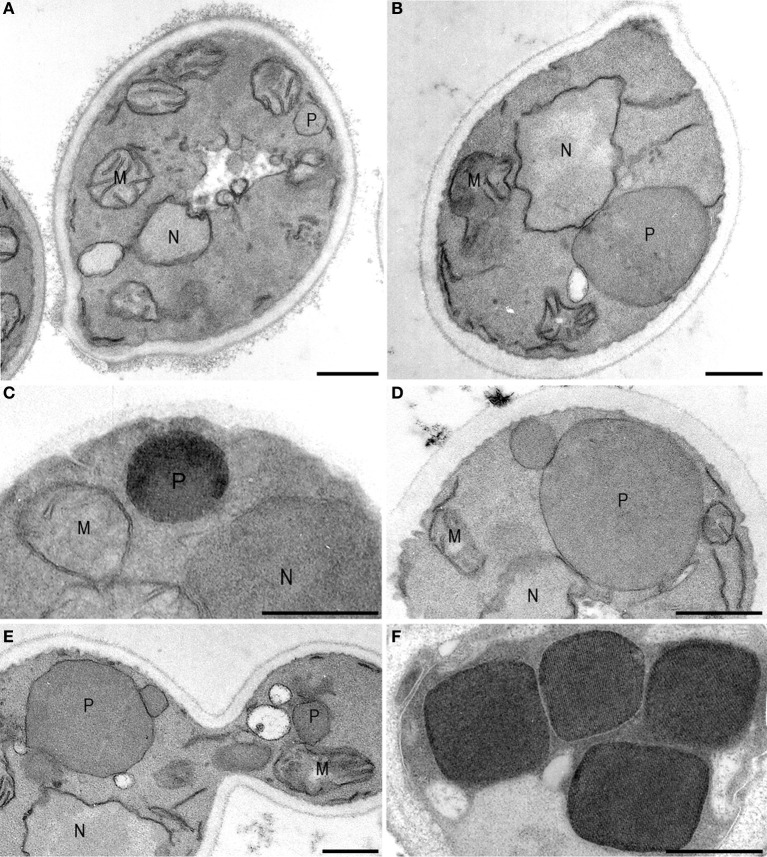
**Peroxisome multiplication imaged**. *Hansenula polymorpha* cells are shifted from glucose- to methanol media, conditions that require peroxisome functions for growth. On glucose, characteristically a single peroxisome is present per cell **(A)** that—upon a shift to methanol containing media—incorporates alcohol oxidase **(C)**, catalase and dihydroxyacetone synthase proteins, which are essential for growth on methanol. As a result the organelle increase in size **(B)** and, after maturation, forms a new organelle by fission **(D)** that subsequently will grow. A similar a-symmetric fission machinery is responsible for the administration of small organelles to the yeast bud **(E)**. When the culture has reached the late exponential phase of growth, typically 4–6 organelles are present of comparable shape. The cuboid shape is due to the presence of large luminal alcohol oxidase crystals. M, mitochondria; N, nucleus; P, peroxisome. Cells are fixed with KMnO4, except **(C,F)**, which are glutaraldehyde fixed. In these cells alcohol oxidase activity is demonstrated using CeCl3.

The origin of peroxisomes is still controversial. Following their discovery, the organelles were considered to bud from the ER based on the observations that peroxisomes—and in particular young developing ones—were invariably seen in close contact with the ER. Since direct contacts between the organelles were not observed, the ER theory was replaced by a model of development by growth and fission of pre-existing ones (Lazarow and Fujiki, 1985). The first morphological data that suggested growth and fission came from kinetic studies using the yeast *Hansenula polymorpha*, shifted from glucose to methanol, conditions that require peroxisome enzymes for growth (Veenhuis et al., 1978). During growth on glucose, *H. polymorpha* cells contain a single peroxisome. In the first 6–8 h after the shift alcohol oxidase and catalase, key peroxisomal enzymes of methanol metabolism, are synthesized and incorporate in the original organelles present in the glucose inoculum cells. After maturation, the organelle formed an extension that subsequently budded off and in turn grew (Figure 1). This way the cells formed 5–7 organelles of approximately equal size (Figure 1) within a period of 24 h of growth. These morphological data were subsequently reinforced by biochemical data which indicated that peroxisomal matrix proteins were synthesized on free ribosomes in the cytosol (Goldman and Blobel, 1978; Fujiki et al., 1985) and post-translationally incorporated in the organelle by a unique protein translocation machinery (Lazarow and Fujiki, 1985).

The first genetic support for the growth and fission model came from studies in the Tabak group, who identified the *Saccharomyces cerevisiae* dynamin-like protein (DLPs) Vps1 as a component involved in peroxisome fission together with actin and the class V myosin motor Myo2 being required for transport of newly separated organelles to the developing bud (Hoepfner et al., 2001). Studies in other yeast species, plant and mammals resolved another DLPs (designated Drp1, Dnm1 or DRP3 respectively) that is involved in peroxisome fission (Koch et al., 2003; Kuravi et al., 2006; Zhang and Hu, 2009). Interestingly, these proteins also play a role in mitochondrial fission. Subsequent studies identified additional components, namely the tail anchored protein Fis1 that, together with Mdv1 (only in yeast) and, unique for *S. cerevisiae*, Caf1, are required to bind Dnm1—but not Vps1—to the target membrane (Motley et al., 2008; Nagotu et al., 2008a). In addition, Mff1 and GDAP1 have been identified to control both mitochondria and peroxisome fission in mammalian cells (Gandre-Babbe and van der Bliek, 2008; Huber et al., 2013). With this, consensus had been reached in the field for the autonomous nature of peroxisomes for many years.

This view changed again when the first data came available on the reintroduction of peroxisomes in cells lacking the organelles due to a mutation in a gene essential for peroxisome membrane biogenesis. Bulk of these studies were conducted with *pex3* or *pex19* cells, in which the organelles reappeared after reintroduction of the corresponding deleted gene, by a process often referred to as “*de novo* peroxisome formation” (Hoepfner et al., 2005; Kragt et al., 2005; Tam et al., 2005; Haan et al., 2006). It is commonly accepted that peroxisomes, which form *de novo*, are not created from scratch, but originate from another membrane in line with the proposition “Omnis membrana e membrana” (Günther Blobel, Nobel Prize 1999). Most of the available experimental data point to the ER as a template for this pathway.

With this, the question raised whether and in how far this process contributes to the total peroxisome population in WT cells. Indications for this came from studies in which *VPS1* and/or *DNM1* were deleted. In all yeast species studied this resulted in the reduction of peroxisome numbers to generally only one organelle per cell. Under these conditions the *de novo* peroxisome formation machinery is normally active since Dnm1 and Vps1 are not involved in this process (Motley et al., 2008; Nagotu et al., 2008b). Very recently, it was shown that a peroxisome-deficient phenotype was obtained in mutant yeast cells in which both *de novo* synthesis and fission are blocked (in *H. polymorpha pex11 pex25* cells), but not when only one of these processes was blocked in *pex11* or *pex25* cells; (Saraya et al., 2011). This reinforces that in yeast the cellular peroxisome population can be maintained predominantly by fission (in *pex25* cells).

Taken all data together, fission appears to be the dominant mode of organelle maintenance in yeast although it cannot be excluded that few organelles are formed *de novo* too in WT cells. Evidence for the latter is however not observed. Possibly, the yeast model is not universal as data have been presented suggesting that in mammals *de novo* synthesis prevails in organelle formation (Kim et al., 2006). However, other studies indicate that mammalian peroxisomes also predominantly form by fission (Huybrechts et al., 2009; Delille et al., 2010).

**Figure 2:**
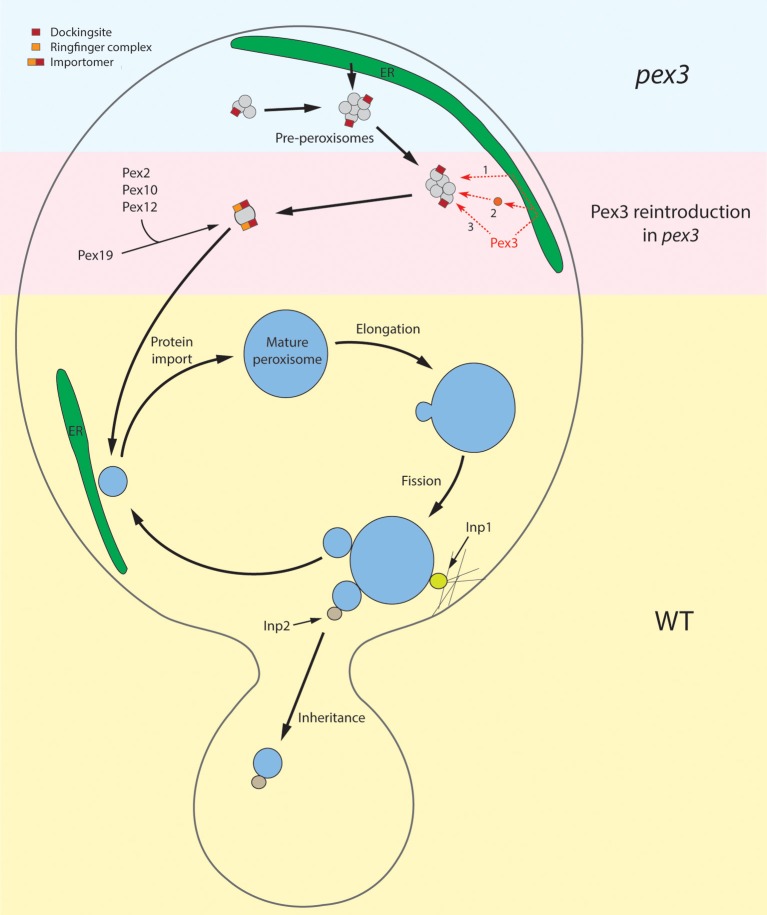
**Schematic overview of peroxisome re-introduction in *pex3* and peroxisome proliferation in WT cells**. The upper part of the cell represents the situation in *pex3* cells. These cells contain vesicular structures that harbor Pex13 and Pex14, proteins of the receptor docking site. How these proteins are sorted to these membranes is unknown. These structures may derive by proliferation of pre-existing ones or be formed from the ER. The pre-peroxisomes are the target for re-introduced Pex3, which may reach these structures via the ER (1), upon incorporation in vesicles that derive from the ER and subsequently fuse with pre-peroxisomes (2) or sort directly to them (3). With the presence of Pex3 at the pre-peroxisome, other PMPs can subsequently be taken up via the Pex3/Pex19 machinery. These include the ring finger proteins Pex2, Pex10, and Pex12, leading to the formation of a functional importomer. This allows uptake of matrix proteins resulting in organelle maturation and subsequent multiplication by fission as depicted in the lower half of the picture (WT situation). During growth the organelle is invariably closely associated with strands of ER. During cell fission Inp1 is essential to dock the mother organelle to the cortex whereas Inp2 determines the delivery of newly formed organelles to the bud.

Clearly, organelle multiplication in substrate induced yeast cells serves different functions: in WT cells new organelles will be formed that will stay in the mother cell and mature to support growth on the carbon source that is supplemented for growth (i.e., oleate or methanol). On the other hand, organelles multiply dependent of the cell cycle to administrate new organelles to the daughter cell (Figure 2). While mother organelles are hooked up in the mother cell via the function of Inp1, these newly formed organelles bind Inp2, which is required for binding of the organelle to Myo2 and subsequent transport to the developing bud (Fagarasanu et al., 2005, 2006; Knoblach et al., 2013). This suggests that upon peroxisome fission two types of organelles may form that biochemically differ. Possibly, this is related to the function of Pex19. This suggestion is based on the important observation that Inp2 is not the sole determinant in Myo2 binding in that Inp2 interacts with both Myo2 and Pex19 to serve the function in organelle transport to the bud (Otzen et al., 2012). Therefore, it may well be that the availability of Pex19 at the membrane (and thus Inp2 binding) is the key determinant that prescribes which organelle is donated to the bud and which one will stay in the mother cell to serve a function in optimal cell metabolism.

Obviously, peroxisome fission and partition have to be carefully controlled to sustain optimal cell health. Indeed, a mutation in human DLP1 results in a lethal phenotype (Waterham et al., 2007). The fission process can be divided into three steps, namely the initial organelle elongation step, growth and constriction of the elongated organelle followed by the actual fission process. Various proteins have been suggested to be involved in peroxisome fission, including members of the Pex11 and the Pex23 protein families (Kiel et al., 2006). In yeast the molecular function of most of these proteins is unknown except for Pex11 and Pex25 (Saraya et al., 2011). Opalinski and colleagues demonstrated that Pex11 is specifically involved in the initial membrane elongation process, a function that is mediated by an amphipathic α-helix located in the N-terminus of the protein and that is conserved between species (Opalinski et al., 2011). The principles of constriction are unknown but may, similar to mitochondria, be related to the function of the ER in conjunction with actin filaments. Scission is mediated by Dlp's, i.e., Vps1 and Dnm1 in baker's yeast (Hoepfner et al., 2001; Kuravi et al., 2006) and Dnm1 in *H. polymorpha* (Nagotu et al., 2008b).

Surprisingly, peroxisome fission is associated with a major rearrangement of various peroxisomal membrane proteins (PMPs) belonging to the importomer that are specifically donated to the developing small organelle (Cepinska et al., 2011). This process appeared to be dependent on the function of Pex11. In contrast to the bulk administration of PMPs to the small peroxisome, fluorescence microscopy analysis revealed that generally very low levels of matrix components, often below the limit of detection, are included in these structures. Apparently, during yeast fission the developing bud is administrated with new organelles that are optimally equipped for their function in matrix protein import (thereby determining their future function!) rather than having an immediate function in cell metabolism (Figure 2).

Obviously, peroxisome development requires functional interactions with other organelles, such as the ER, where most peroxisomal phospholipids are synthesized (Raychaudhuri and Prinz, 2008) as well as with mitochondria, which synthesize cardiolipin, a lipid present in the peroxisomal membrane (Wriessnegger et al., 2007), and heme (for catalase synthesis). So far, very little is known on possible physical interactions of peroxisomes and other organelles, which may contribute to various aspects of peroxisome proliferation.

## *De novo* peroxisome formation

### Peroxisome reintroduction in *pex3* yeast cells

The most extensively used experimental system to study *de novo* peroxisome formation in yeast is the reintroduction of peroxisomes in *pex3* strains in which a *PEX3-GFP* hybrid gene is placed under control of an inducible promoter (Hoepfner et al., 2005; Kragt et al., 2005; Tam et al., 2005; Haan et al., 2006). Fluorescence microscopy (FM) analysis revealed that upon induction newly synthesized Pex3-GFP sorts to the ER, concentrates in foci followed by the formation of a pre-peroxisomal structure, which pinches off and develops into a nascent peroxisome. Alternatively two (*S. cerevisiae*; van der Zand et al., 2012) or multiple (*Y. lipolytica*; Titorenko et al., 2000) types of vesicles have been proposed to pinch off from the ER, which subsequently fuse to form a nascent peroxisome.

Invariably, these models predict that Pex3 initially sorts to the ER. This is strengthened by the finding that the extreme N-terminus of Pex3 proteins has characteristics in common with signal-anchor-like sequences, typical for authentic ER membrane proteins (Thoms et al., 2012; Fakieh et al., 2013). Another argument for ER sorting of Pex3 is the observation that peroxisome reintroduction in *S. cerevisiae pex3* cells is affected in a temperature sensitive *sec61* mutant strain or upon depletion of Sec61 (Thoms et al., 2012). However, opposite data were described by the Gould group (South et al., 2001) using a cold-sensitive *S. cerevisiae sec61* mutant.

Also, experiments reported by Kragt and colleagues suggest initial sorting of Pex3 to the ER (Kragt et al., 2005). In this study a Pex3 variant was used containing an artificial ER signal peptide and cleavage site, which functionally complemented *S. cerevisiae pex3* cells, whereas a similar construct in which cleavage of the signal peptide was blocked by a mutation did not. This result was interpreted by an inability of the uncleaved variant to exit the ER during *de novo* peroxisome formation.

However, these studies and other studies were conducted at conditions in which the N-terminus of Pex3 was modified or in which *PEX3* was not under control of its endogenous promoter, which could lead to artificial mislocalization.

Analysis of mRNA transcript levels demonstrated that the expression of genes encoding peroxins/PMPs hardly increased at peroxisome induction conditions (Kal et al., 1999). Hence, overproduction is readily achieved which may lead to mislocalization. This may also be true for Pex3, when produced under control of strong inducible promoters, even when the ultimate Pex3 levels do not exceed WT levels. This is related to the fact that the initial kinetics of the P_GAL_ driven Pex3 synthesis strongly exceeds those normally occurring in WT cells via the endogenous promoter.

Indeed, also data have been presented suggesting that the ER is not the target membrane for authentic Pex3. For instance, studies by Matsuzaki and Fujiki, who analyzed *in vitro* insertion of Pex3 in different cellular fractions of Chinese hamster ovary cells, revealed that Pex3 only inserted in peroxisomal membranes, but not in microsomes or mitochondria (Matsuzaki and Fujiki, 2008). Similarly, *in vitro* experiments indicated that PMP24 was directly inserted in rat liver peroxisomes in a Pex3 and Pex19 dependent manner (Pinto et al., 2006).

Although the data reported by Kragt and colleagues, strongly suggest that Pex3 traffics via the ER (Kragt et al., 2005), a similar approach in the Erdmann group using a Pex3 variant containing artificial sorting information for the mitochondrial outer membrane protein, also resulted in functional complementation of *pex3* cells (Rucktaschel et al., 2010). This at least suggests that Pex3 not necessarily needs to sort via the ER for *de novo* peroxisome formation. Moreover, for both experiments it cannot be excluded that a minor portion of the modified Pex3 escapes from sorting to mitochondria or ER.

Moreover, two recent studies revealed that newly synthesized Pex3 targets to all pre-existing peroxisomes in WT yeast cells (Fakieh et al., 2013; Menendez-Benito et al., 2013). If under these conditions Pex3 would traffic via the ER, a vesicular transport pathway from the ER to pre-existing peroxisomes should exist. Indeed data have been presented supporting this possibility (Motley and Hettema, 2007). On the other hand, to the best of our knowledge Pex3-GFP has never been localized to the ER in WT cells.

As is clear from the above, both data indicating that Pex3 directly sorts to peroxisomes or traffics via the ER to these organelles have been presented. A model explaining these seemingly contradicting observations could be that both at the ER and peroxisomes a Pex3 insertion machinery exists. Assuming that the ER insertion machinery has a lower affinity for newly synthesized Pex3 relative to the peroxisomal one, Pex3 will predominantly sort to the high affinity site at peroxisomes in WT cells. However, in cells lacking peroxisomes or at Pex3 overproduction conditions Pex3 will then (also) be sorted to the ER.

*In vitro* protein insertion studies as well as *in vivo* experiments tracking (single) Pex3 molecules on their way to peroxisomes in WT cells (e.g., in pulse chase experiments and/or superresolution fluorescence microscopy), will help to resolve this urgent question.

### pex3 dependent peroxisome reintroduction: an alternative pathway

We recently re-investigated the location of Pex14 in *H. polymorpha pex3* cells using high resolution immune electron microscopy and observed that the Pex14-GFP spots in fact represent vesicular structures located at the vicinity of mitochondria or ER, but never were found connected to these organelles. Comparable observations were made using *H. polymorpha pex19* cells (Knoops et al., 2014). Using deconvolution microscopy, such structures previously were also observed in *P. pastoris pex3* cells.

These recent studies revealed that the Pex14-containing structures in *H. polymorpha pex3* cells contained, besides Pex14, also Pex8 and Pex13 and hence may contain a functional receptor docking site. However, none of the other PMPs tested (Pex10, Pmp47, Pex11) was observed at these structures, so a functional importomer was not formed. Hence, similar to matrix protein import mutants, *pex3* cells apparently contain peroxisome ghosts that do not harbor all typical marker PMPs, but only a subset. This may add to the explanation why they were overlooked in earlier studies. The origin of these structures is currently unknown. We speculate that they could proliferate from pre-existing structures, like assumed for other peroxisomal ghosts. Alternatively, they may form from the ER (Figure 2). If so, however, their formation is independent of Pex3.

Interestingly, the Pex14-containing structures, but not the ER, were the target for reintroduced Pex3-GFP after which these structures developed into normal functional peroxisomes. Moreover, we also observed that Pex25 and Pex19, two other peroxins proposed to be involved in the *de novo* peroxisome formation, are not involved in the formation of the vesicles in *pex3* cells. This new insight into peroxisome formation in *pex3* cells fundamentally differs from the generally accepted models and may stimulate further studies on the principles of *de novo* peroxisome formation.

### Peroxisome reintroduction in inheritance mutants

Cells of mutants defective in peroxisome segregation (*inp1* or *inp2* deletion strains) temporarily lack peroxisomal structures detectable by FM (Fagarasanu et al., 2005, 2006). In these cells peroxisomes reappear shortly after budding is completed, presumably by *de novo* peroxisome formation (Motley and Hettema, 2007). So far, the reappearance of peroxisomes in *inp1* or *inp2* cells has only been studied using matrix marker proteins, but not PMP markers. Hence, it cannot be excluded yet that in these cells also small peroxisomal remnants occur, like in *pex3* cells. It is important to solve this issue, using PMP marker proteins also including Pex3. If ghosts are fully absent, these mutants would be the preferred model systems for analyzing *de novo* peroxisome formation.

Interestingly, upon deletion of *PEX25* in *S. cerevisiae inp2* cells, the cells become peroxisome deficient, indicating that the *de novo* peroxisome formation process is blocked in these cells (Huber et al., 2012). Pex25 is also required for *de novo* formation in yeast *pex3* cells upon reintroduction of the *PEX3* gene (Saraya et al., 2011; Huber et al., 2012). These data indicate that both *de novo* peroxisome formation processes depend on Pex25 and probably represent the same process.

### The disputed PMP sorting machinery

As for the role of the ER in *de novo* peroxisome formation, also no consensus is reached on the involvement of the ER in trafficking of PMPs other than Pex3 to peroxisomes in WT cells. Current models range from PMP trafficking via the ER to direct post-translational insertion in peroxisomal membranes (Schliebs and Kunau, 2004; Menendez-Benito et al., 2013; Yagita et al., 2013). In the first model Pex3 and Pex19 play a role in the exit of PMPs from the ER. In the second one Pex19 serves as a soluble receptor/chaperone that binds newly synthesized PMPs and is recruited to the peroxisomal membrane by Pex3 (Fang et al., 2004), followed by the insertion of PMPs by a yet unknown mechanism. According to this model PMPs are predicted to be cytosolic or mistargeted to other cellular membranes in the absence of Pex3 or Pex19. The fact that many PMPs physically interact with Pex19 strongly supports the second model. Also, the observations that the levels of many PMPs strongly drop in *pex3* cells, and often are below the limit of detection, are in favor of this model. However, also many data in support of the first model have been presented.

van der Zand and colleagues determined the localization of 16 PMPs using fluorescence microscopy upon pulsed induction using P_GAL_ which suggested that these proteins initially sorted to the ER (van der Zand et al., 2010). As indicated above, these experiments should be interpreted with care because of the strong, initial temporal overexpression due to using P_GAL_. Moreover, also in these studies the limitations of the relatively low resolution of fluorescence microscopy can easily result in misinterpretation of the data. The same authors also analyzed the localization of PMPs in *pex3* cells and concluded that all accumulated at the ER. Careful re-inspection of the published images suggests that Pex8, Pex13 and Pex14 indeed were present in foci, whereas the other PMPs tested (Pex2, Pex6, Pex11, Pex15) showed a very low, dispersed localization. We recently observed that also *S. cerevisiae pex3* cells harbor Pex14-containing peroxisomal ghosts, like in *H. polymorpha pex3* cells (unpublished results). Hence, most likely also in *S. cerevisiae* Pex8, Pex13 and Pex14 are present at peroxisomal membrane structures, whereas the other PMPs are instable and located to the cytosol.

In line with initial ER sorting would be a role of the Sec complex in PMP routing. Indeed, upon *in vivo* depletion of Sec components, a portion of certain PMPs became soluble (van der Zand et al., 2010). Also, data have been presented showing that peroxisomal tail anchor proteins depend on the function of the Get complex. For instance, Schuldiner and colleagues showed that the tail anchored protein Pex15 mislocalized to mitochondria when a component of the GET complex was depleted. Moreover, a physical interaction between Pex15 and Get complex components has been demonstrated (Schuldiner et al., 2008). Conversely, however, the insertion of the mammalian homolog of Pex15, Pex26, depends of Pex19 (Halbach et al., 2006; Matsuzono and Fujiki, 2006) and is independent of TRC40, the mammalian homolog of Get2 (Yagita et al., 2013). Also, insertion of the tail anchored protein Fis1 in peroxisomal membranes was shown to depend on Pex19 (Delille and Schrader, 2008).

As argued before for *pex3* cells, in fact both pathways may exist simultaneously. In this view the final location of the PMP is determined by the affinity of it targeting information for either the ER or normal peroxisomes. An alternative may be that—at least in part—different pathways exist, depending on marker protein and model organism used.

For instance, our recent findings clearly show that the localization of Pex13 and Pex14 into peroxisomal membranes does not require Pex3. However, in the same cells other PMPs require the Pex3/Pex19 machinery for stability and insertion in these membranes. This is supported by various data previously reported for *S. cerevisiae* and *P. pastoris* (Hettema et al., 2000; Hazra et al., 2002).

Interestingly, also in human cells Pex13 was shown to be able to insert into peroxisomal membranes independent of Pex19, whereas in yeast and mammals Pex13 is essential for the association of Pex14 with the peroxisomal membrane (Fransen et al., 2004). These data underscore that these two peroxins do not require the Pex3/Pex19 machinery for proper membrane insertion.

## Conclusions

Peroxisome proliferation at inducing conditions is heavily debated but in yeast consensus is achieved that in these organisms fission is the main mode of organelle multiplication rather than *de novo* synthesis. Also in mammals the major mode of peroxisome proliferation is most likely fission, although *de novo* synthesis may occur as well.

*De novo* synthesis in yeast is observed in cells that lack peroxisomes. This process is in particular studied in *pex3* strains upon reintroduction of the *PEX3* gene. Recent data however indicated that *pex3* cells contain peroxisomal vesicles that form in the absence of Pex3 (Knoops et al., 2014). It was shown that in *pex3* cells not the ER but in fact these peroxisomal vesicles were the target for Pex3 and the subsequent formation of peroxisomes. However, various questions remain. For instance, it is unknown where the vesicles in *pex3* cells originate from. They may arise by fission of existing structures but also form from the ER (Figure 2). In the latter view these *in vivo* data may complement recent *in vitro* studies in which pre-peroxisomes were formed from microsomal fractions (Lam et al., 2011) or in permeabilized cells (Agrawal et al., 2011). Clearly, the *in vivo* data suggest a novel concept of peroxisome reintroduction in *pex3* cells and as such may promote future studies in this field. One approach may involve searching for novel proteins involved in *de novo* synthesis. A recent model that genetically separates *de novo* synthesis from fission may be useful in this respect. Two independent studies convincingly showed that mutants affected in fission or *de novo* synthesis do not display a peroxisome-deficient phenotype (Saraya et al., 2011; Huber et al., 2012). Only the combination of the two mutations as in a *pex11 pex25* double mutant leads to the absence of peroxisomes. With this, an elegant screen is now available for identifying novel components involved in *de novo* synthesis by creating double mutants in a *pex11* strain and select for peroxisome-deficient mutants.

Finally, the principles of PMP sorting are far from solved and change from the view that all PMPs travel via the ER to the assumption that PMPs travel directly to the target organelle. Considering the current literature, it is likely that both pathways in fact exist simultaneously. In this respect it is relevant to study the effect of manipulating modulation the affinity of the two sorting signals proposed (either for the ER or the intact peroxisome) for their substrate organelle. This may help in understanding why the protein travels to the ER in peroxisome-deficient mutants but to the intact organelle at WT conditions.

So far, most FM approaches used suffer from distinct drawbacks (i.e., overexpression effects) that do not allow drawing unequivocal conclusions for WT conditions. Clearly, novel techniques are required, like pulse chase experiments to show the transient ER location of specific PMPs in conjunctions with high speed microscopy techniques to track the routing of these proteins.

### Conflict of interest statement

The authors declare that the research was conducted in the absence of any commercial or financial relationships that could be construed as a potential conflict of interest.

## References

[B1] Agrawal G. Joshi S. Subramani S. (2011). Cell-free sorting of peroxisomal membrane proteins from the endoplasmic reticulum. Proc. Natl. Acad. Sci. U.S.A. 108, 9113–9118 10.1073/pnas.1018749108 21576455 PMC3107335

[B2] Cepinska M. N. Veenhuis M. van der Klei I. J. Nagotu S. (2011). Peroxisome fission is associated with reorganization of specific membrane proteins. Traffic 12, 925–937 10.1111/j.1600-0854.2011.01198.x 21507161

[B3] Delille H. K. Agricola B. Guimaraes S. C. Borta H. Luers G. H. Fransen M. (2010). Pex11pbeta-mediated growth and division of mammalian peroxisomes follows a maturation pathway. J. Cell Sci. 123, 2750–2762 10.1242/jcs.062109 20647371

[B4] Delille H. K. Schrader M. (2008). Targeting of hFis1 to peroxisomes is mediated by Pex19p. J. Biol. Chem. 283, 31107–31115 10.1074/jbc.M803332200 18782765 PMC2662177

[B5] Fagarasanu A. Fagarasanu M. Eitzen G. A. Aitchison J. D. Rachubinski R. A. (2006). The peroxisomal membrane protein Inp2p is the peroxisome-specific receptor for the myosin V motor Myo2p of *Saccharomyces cerevisiae*. Dev. Cell 10, 587–600 10.1016/j.devcel.2006.04.012 16678774

[B6] Fagarasanu M. Fagarasanu A. Tam Y. Y. Aitchison J. D. Rachubinski R. A. (2005). Inp1p is a peroxisomal membrane protein required for peroxisome inheritance in *Saccharomyces cerevisiae*. J. Cell Biol. 169, 765–775 10.1083/jcb.200503083 15928207 PMC2171609

[B7] Fakieh M. H. Drake P. J. Lacey J. Munck J. M. Motley A. M. Hettema E. H. (2013). Intra-ER sorting of the peroxisomal membrane protein Pex3 relies on its luminal domain. Biol. Open 2, 829–837 10.1242/bio.20134788 23951409 PMC3744075

[B8] Fang Y. Morrell J. C. Jones J. M. Gould S. J. (2004). PEX3 functions as a PEX19 docking factor in the import of class I peroxisomal membrane proteins. J. Cell Biol. 164, 863–875 10.1083/jcb.200311131 15007061 PMC2172291

[B9] Fransen M. Vastiau I. Brees C. Brys V. Mannaerts G. P. Van Veldhoven P. P. (2004). Potential role for Pex19p in assembly of PTS-receptor docking complexes. J. Biol. Chem. 279, 12615–12624 10.1074/jbc.M304941200 14715663

[B10] Fujiki Y. Rachubinski R. A. Mortensen R. M. Lazarow P. B. (1985). Synthesis of 3-ketoacyl-CoA thiolase of rat liver peroxisomes on free polyribosomes as a larger precursor. Induction of thiolase mRNA activity by clofibrate. Biochem. J. 226, 697–704 3985942 10.1042/bj2260697 PMC1144767

[B11] Gandre-Babbe S. van der Bliek A. M. (2008). The novel tail-anchored membrane protein Mff controls mitochondrial and peroxisomal fission in mammalian cells. Mol. Biol. Cell 19, 2402–2412 10.1091/mbc.E07-12-1287 18353969 PMC2397315

[B12] Goldman B. M. Blobel G. (1978). Biogenesis of peroxisomes: intracellular site of synthesis of catalase and uricase. Proc. Natl. Acad. Sci. U.S.A. 75, 5066–5070 10.1073/pnas.75.10.5066 368807 PMC336264

[B13] Haan G. J. Baerends R. J. Krikken A. M. Otzen M. Veenhuis M. van der Klei I. J. (2006). Reassembly of peroxisomes in *Hansenula polymorpha* pex3 cells on reintroduction of Pex3p involves the nuclear envelope. FEMS Yeast Res. 6, 186–194 10.1111/j.1567-1364.2006.00037.x 16487342

[B14] Halbach A. Landgraf C. Lorenzen S. Rosenkranz K. Volkmer-Engert R. Erdmann R. (2006). Targeting of the tail-anchored peroxisomal membrane proteins PEX26 and PEX15 occurs through C-terminal PEX19-binding sites. J. Cell Sci. 119, 2508–2517 10.1242/jcs.02979 16763195

[B15] Hazra P. P. Suriapranata I. Snyder W. B. Subramani S. (2002). Peroxisome remnants in pex3delta cells and the requirement of Pex3p for interactions between the peroxisomal docking and translocation subcomplexes. Traffic 3, 560–574 10.1034/j.1600-0854.2002.30806.x 12121419

[B16] Hettema E. H. Girzalsky W. van den Berg M. Erdmann R. Distel B. (2000). *Saccharomyces cerevisiae* pex3p and pex19p are required for proper localization and stability of peroxisomal membrane proteins. EMBO J. 19, 223–233 10.1093/emboj/19.2.223 10637226 PMC305556

[B17] Hoepfner D. Schildknegt D. Braakman I. Philippsen P. Tabak H. F. (2005). Contribution of the endoplasmic reticulum to peroxisome formation. Cell 122, 85–95 10.1016/j.cell.2005.04.025 16009135

[B18] Hoepfner D. van den Berg M. Philippsen P. Tabak H. F. Hettema E. H. (2001). A role for Vps1p, actin, and the Myo2p motor in peroxisome abundance and inheritance in *Saccharomyces cerevisiae*. J. Cell Biol. 155, 979–990 10.1083/jcb.200107028 11733545 PMC2150915

[B19] Huber A. Koch J. Kragler F. Brocard C. Hartig A. (2012). A subtle interplay between three Pex11 proteins shapes *de novo* formation and fission of peroxisomes. Traffic 13, 157–167 10.1111/j.1600-0854.2011.01290.x 21951626 PMC3245845

[B20] Huber N. Guimaraes S. Schrader M. Suter U. Niemann A. (2013). Charcot-Marie-Tooth disease-associated mutants of GDAP1 dissociate its roles in peroxisomal and mitochondrial fission. EMBO Rep. 14, 545–552 10.1038/embor.2013.56 23628762 PMC3674444

[B21] Huybrechts S. J. Van Veldhoven P. P. Brees C. Mannaerts G. P. Los G. V. Fransen M. (2009). Peroxisome dynamics in cultured mammalian cells. Traffic 10, 1722–1733 10.1111/j.1600-0854.2009.00970.x 19719477

[B22] Kal A. J. van Zonneveld A. J. Benes V. van den Berg M. Koerkamp M. G. Albermann K. (1999). Dynamics of gene expression revealed by comparison of serial analysis of gene expression transcript profiles from yeast grown on two different carbon sources. Mol. Biol. Cell 10, 1859–1872 10.1091/mbc.10.6.1859 10359602 PMC25383

[B23] Kiel J. A. Veenhuis M. van der Klei I. J. (2006). PEX genes in fungal genomes: common, rare or redundant. Traffic 7, 1291–1303 10.1111/j.1600-0854.2006.00479.x 16978390

[B24] Kim P. K. Mullen R. T. Schumann U. Lippincott-Schwartz J. (2006). The origin and maintenance of mammalian peroxisomes involves a *de novo* PEX16-dependent pathway from the ER. J. Cell Biol. 173, 521–532 10.1083/jcb.200601036 16717127 PMC2063862

[B25] Knoblach B. Sun X. Coquelle N. Fagarasanu A. Poirier R. L. Rachubinski R. A. (2013). An ER-peroxisome tether exerts peroxisome population control in yeast. EMBO J. 32, 2439–2453 10.1038/emboj.2013.170 23900285 PMC3770948

[B26] Knoops K. Manivannan S. Cepińska M. N. Krikken A. M. Kram A. M. Veenhuis M. (2014). Preperoxisomal vesicles can form in the absence of Pex3. J. Cell Biol. 204, 659–668 10.1083/jcb.201310148 24590171 PMC3941047

[B27] Koch A. Thiemann M. Grabenbauer M. Yoon Y. Mcniven M. A. Schrader M. (2003). Dynamin-like protein 1 is involved in peroxisomal fission. J. Biol. Chem. 278, 8597–8605 10.1074/jbc.M211761200 12499366

[B28] Kragt A. Voorn-Brouwer T. van den Berg M. Distel B. (2005). Endoplasmic reticulum-directed Pex3p routes to peroxisomes and restores peroxisome formation in a *Saccharomyces cerevisiae* pex3Delta strain. J. Biol. Chem. 280, 34350–34357 10.1074/jbc.M505432200 16100114

[B29] Kuravi K. Nagotu S. Krikken A. M. Sjollema K. Deckers M. Erdmann R. (2006). Dynamin-related proteins Vps1p and Dnm1p control peroxisome abundance in *Saccharomyces cerevisiae*. J. Cell Sci. 119, 3994–4001 10.1242/jcs.03166 16968746

[B30] Lam S. K. Yoda N. Schekman R. (2011). A vesicle carrier that mediates peroxisome protein traffic from the endoplasmic reticulum. Proc. Natl. Acad. Sci. U.S.A. 108, E51–E52 10.1073/pnas.1103526108 21467226 PMC3078416

[B31] Lazarow P. B. Fujiki Y. (1985). Biogenesis of peroxisomes. Annu. Rev. Cell Biol. 1, 489–530 10.1146/annurev.cb.01.110185.002421 3916321

[B32] Matsuzaki T. Fujiki Y. (2008). The peroxisomal membrane protein import receptor Pex3p is directly transported to peroxisomes by a novel Pex19p- and Pex16p-dependent pathway. J. Cell Biol. 183, 1275–1286 10.1083/jcb.200806062 19114594 PMC2606968

[B33] Matsuzono Y. Fujiki Y. (2006). *In vitro* transport of membrane proteins to peroxisomes by shuttling receptor Pex19p. J. Biol. Chem. 281, 36–42 10.1074/jbc.M509819200 16280322

[B34] Menendez-Benito V. van Deventer S. J. Jimenez-Garcia V. Roy-Luzarraga M. van Leeuwen F. Neefjes J. (2013). Spatiotemporal analysis of organelle and macromolecular complex inheritance. Proc. Natl. Acad. Sci. U.S.A. 110, 175–180 10.1073/pnas.1207424110 23248297 PMC3538235

[B35] Motley A. M. Hettema E. H. (2007). Yeast peroxisomes multiply by growth and division. J. Cell Biol. 178, 399–410 10.1083/jcb.200702167 17646399 PMC2064844

[B36] Motley A. M. Ward G. P. Hettema E. H. (2008). Dnm1p-dependent peroxisome fission requires Caf4p, Mdv1p and Fis1p. J. Cell Sci. 121, 1633–1640 10.1242/jcs.026344 18445678 PMC2579327

[B37] Nagotu S. Krikken A. M. Otzen M. Kiel J. A. Veenhuis M. van der Klei I. J. (2008a). Peroxisome fission in *Hansenula polymorpha* requires Mdv1 and Fis1, two proteins also involved in mitochondrial fission. Traffic 9, 1471–1484 10.1111/j.1600-0854.2008.00772.x 18513378

[B38] Nagotu S. Saraya R. Otzen M. Veenhuis M. van der Klei I. J. (2008b). Peroxisome proliferation in *Hansenula polymorpha* requires Dnm1p which mediates fission but not *de novo* formation. Biochim. Biophys. Acta 1783, 760–769 10.1016/j.bbamcr.2007.10.018 18060881

[B39] Opalinski L. Kiel J. A. Williams C. Veenhuis M. van der Klei I. J. (2011). Membrane curvature during peroxisome fission requires Pex11. EMBO J. 30, 5–16 10.1038/emboj.2010.299 21113128 PMC3020119

[B40] Otzen M. Ruckt R. Thoms S. Emmrich K. Krikken A. M. Erdmann R. (2012). Pex19p contributes to peroxisome inheritance in the association of peroxisomes to Myo2p. Traffic 13, 947–959 10.1111/j.1600-0854.2012.01364.x 22486971

[B41] Pinto M. P. Grou C. P. Alencastre I. S. Oliveira M. E. Sa-Miranda C. Fransen M. (2006). The import competence of a peroxisomal membrane protein is determined by Pex19p before the docking step. J. Biol. Chem. 281, 34492–34502 10.1074/jbc.M607183200 16980692

[B42] Raychaudhuri S. Prinz W. A. (2008). Nonvesicular phospholipid transfer between peroxisomes and the endoplasmic reticulum. Proc. Natl. Acad. Sci. U.S.A. 105, 15785–15790 10.1073/pnas.0808321105 18836080 PMC2572964

[B43] Rucktaschel R. Halbach A. Girzalsky W. Rottensteiner H. Erdmann R. (2010). *De novo* synthesis of peroxisomes upon mitochondrial targeting of Pex3p. Eur. J. Cell Biol. 89, 947–954 10.1016/j.ejcb.2010.06.012 20655617

[B44] Saraya R. Krikken A. M. Veenhuis M. van der Klei I. J. (2011). Peroxisome reintroduction in *Hansenula polymorpha* requires Pex25 and Rho1. J. Cell Biol. 193, 885–900 10.1083/jcb.201012083 21606207 PMC3105547

[B45] Schliebs W. Kunau W. H. (2004). Peroxisome membrane biogenesis: the stage is set. Curr. Biol. 14, R397–R399 10.1016/j.cub.2004.05.017 15186768

[B46] Schuldiner M. Metz J. Schmid V. Denic V. Rakwalska M. Schmitt H. D. (2008). The GET complex mediates insertion of tail-anchored proteins into the ER membrane. Cell 134, 634–645 10.1016/j.cell.2008.06.025 18724936 PMC2572727

[B47] South S. T. Baumgart E. Gould S. J. (2001). Inactivation of the endoplasmic reticulum protein translocation factor, Sec61p, or its homolog, Ssh1p, does not affect peroxisome biogenesis. Proc. Natl. Acad. Sci. U.S.A. 98, 12027–12031 10.1073/pnas.221289498 11593013 PMC59761

[B48] Tam Y. Y. Fagarasanu A. Fagarasanu M. Rachubinski R. A. (2005). Pex3p initiates the formation of a preperoxisomal compartment from a subdomain of the endoplasmic reticulum in *Saccharomyces cerevisiae*. J. Biol. Chem. 280, 34933–34939 10.1074/jbc.M506208200 16087670

[B49] Thoms S. Harms I. Kalies K. U. Gartner J. (2012). Peroxisome formation requires the endoplasmic reticulum channel protein Sec61. Traffic 13, 599–609 10.1111/j.1600-0854.2011.01324.x 22212716

[B50] Titorenko V. I. Chan H. Rachubinski R. A. (2000). Fusion of small peroxisomal vesicles *in vitro* reconstructs an early step in the *in vivo* multistep peroxisome assembly pathway of *Yarrowia lipolytica*. J. Cell Biol. 148, 29–44 10.1083/jcb.148.1.29 10629216 PMC2156211

[B51] van der Zand A. Braakman I. Tabak H. F. (2010). Peroxisomal membrane proteins insert into the endoplasmic reticulum. Mol. Biol. Cell 21, 2057–2065 10.1091/mbc.E10-02-0082 20427571 PMC2883949

[B52] van der Zand A. Gent J. Braakman I. Tabak H. F. (2012). Biochemically distinct vesicles from the endoplasmic reticulum fuse to form peroxisomes. Cell 149, 397–409 10.1016/j.cell.2012.01.054 22500805

[B53] Veenhuis M. van Dijken J. P. Pilon S. A. Harder W. (1978). Development of crystalline peroxisomes in methanol-grown cells of the yeast *Hansenula polymorpha* and its relation to environmental conditions. Arch. Microbiol. 117, 153–163 10.1007/BF00402303 678021

[B54] Waterham H. R. Koster J. van Roermund C. W. Mooyer P. A. Wanders R. J. Leonard J. V. (2007). A lethal defect of mitochondrial and peroxisomal fission. N. Engl. J. Med. 356, 1736–1741 10.1056/NEJMoa064436 17460227

[B55] Wriessnegger T. Gubitz G. Leitner E. Ingolic E. Cregg J. De La Cruz B. J. (2007). Lipid composition of peroxisomes from the yeast *Pichia pastoris* grown on different carbon sources. Biochim. Biophys. Acta 1771, 455–461 10.1016/j.bbalip.2007.01.004 17293161

[B56] Yagita Y. Hiromasa T. Fujiki Y. (2013). Tail-anchored PEX26 targets peroxisomes via a PEX19-dependent and TRC40-independent class I pathway. J. Cell Biol. 200, 651–666 10.1083/jcb.201211077 23460677 PMC3587837

[B57] Zhang X. Hu J. (2009). Two small protein families, DYNAMIN-RELATED PROTEIN3 and FISSION1, are required for peroxisome fission in Arabidopsis. Plant J. 57, 146–159 10.1111/j.1365-313X.2008.03677.x 18785999

